# Continuing professional development challenges in a rural setting: A mixed-methods study

**DOI:** 10.1007/s40037-022-00718-8

**Published:** 2022-08-09

**Authors:** Melissa Campos-Zamora, Hannah Gilbert, Ramiro I. Esparza-Perez, Melchor Sanchez-Mendiola, Roxane Gardner, Jeremy B. Richards, Mario I. Lumbreras-Marquez, Valerie A. Dobiesz

**Affiliations:** 1 Postgraduate Medical Education, Harvard Medical School, Boston, MA USA; 2 Department of Global Health and Social Medicine, Harvard Medical School, Boston, MA USA; 3 University of Guadalajara, Guadalajara, Mexico; 4 National Autonomous University of Mexico, Mexico City, Mexico; 5 Center for Medical Simulation, Brigham and Women’s Hospital, Boston, MA USA; 6 Shapiro Center for Education and Research, Division of Pulmonary, Critical Care, and Sleep Medicine, Beth Israel Deaconess Medical Center, Boston, MA USA; 7 Department of Anesthesiology, Perioperative and Pain Medicine, Brigham and Women’s Hospital, Boston, MA USA; 8 STRATUS Center for Medical Simulation, Department of Emergency Medicine Brigham and Women’s Hospital, Harvard Humanitarian Initiative, Harvard Medical School, Boston, MA USA

**Keywords:** Continuing professional development, Rural health, Global health, Health profession education

## Abstract

**Introduction:**

Health professionals in rural settings encounter a wide range of medical conditions requiring broad knowledge for their clinical practice. This creates the need for ongoing continuing professional development (CPD). In this study, we explored the barriers that health professionals in a rural healthcare context faced participating in CPD activities and their preferences regarding educational strategies to overcome these challenges.

**Methods:**

This mixed-methods (exploratory sequential) study in a community hospital in rural Mexico includes 22 interviews, 3 focus groups, 40 observational hours, and a questionnaire of healthcare staff.

**Results:**

Despite low engagement with CPD activities (67% not motivated), all participants expressed interest and acknowledged the importance of learning for their practice. Barriers to participating include a disparity between strategies used (lecture-based) and their desire for practical learning, institutional barriers (poor leadership engagement, procedural flaws, and lack of resources), and collaboration barriers (adverse interprofessional education environment, ineffective teamwork, and poor communication). Additional barriers identified were inconvenient scheduling of sessions (75%), inadequate classrooms (65%), high workload (60%), ineffective speakers (60%), and boring sessions (55%). Participants’ preferred learning strategies highlighted activities relevant to their daily clinical activities (practical workshops, simulations, and case analysis). The questionnaire had an 18% response rate.

**Discussion:**

The barriers to CPD in this rural setting are multifactorial and diverse. A strong interest to engage in context-specific active learning strategies highlighted the need for leadership to prioritize interprofessional education, teamwork, and communication to enhance CPD and patient care. These results could inform efforts to strengthen CPD in other rural contexts.

**Supplementary Information:**

The online version of this article (10.1007/s40037-022-00718-8) contains supplementary material, which is available to authorized users.

## Introduction

Continuing professional development (CPD) can positively influence health professionals’ behavior and patient outcomes [1, 2]. However, best practices in health professions education have not been universally translated into CPD practice, and the development and implementation of these strategies vary among different contexts and institutions [3–5]. In rural settings, health professionals often provide the majority of medical care to the population they serve and function as first-contact care for medical emergencies, with their closest referral center hours away. Providers must treat and stabilize patients with injuries and illnesses from a broad range of clinical conditions, requiring broad knowledge. Globally, these professionals encounter a myriad of challenges for CPD due to complex interactions of geographic, historical, sociocultural, and economic factors [6]. Moreover, isolation from academic centers limits their access to educational activities, and qualified instructors are not always readily available [7, 8].

CPD barriers for rural health professionals reported in the literature include communication issues, time constraints, isolated profession-based educational practices, and low priority given to educational activities [9]. Furthermore, educational inequities determined by school, geography, and socioeconomic context may cause health professionals working in rural hospitals to practice extensively with limited opportunities for CPD. Despite these identified challenges there is limited rigorous research on how rural practitioners engage with continuing education, the barriers they face for lifelong learning, and the mechanisms needed to overcome existing obstacles.

We conducted a mixed-methods study among health professionals in a rural healthcare setting with the specific aims to (1) describe the barriers that health professionals in a rural healthcare context face participating in CPD activities and (2) explore preferences regarding educational strategies to overcome these challenges.

## Methods

### Study setting

This study was conducted at “Angel Albino Corzo” (HAAC) community hospital in Chiapas, one of Mexico’s most economically disadvantaged regions, where inadequate health infrastructure has translated into lower health standards [10] and socioeconomic inequalities are replicated in health professions education [11]. This facility provides care for ~ 28,883 inhabitants (in collaboration with an adjacent birth center), with 72 employees. Nursing students from a local school complete their clinical learning requirements in this hospital. However, their curriculum and classes are supervised by their university and not open to the rest of the hospital staff. At the time of this study, there was no formal CPD curriculum in the hospital, with irregular educational sessions being conducted.

### Study design

This exploratory sequential mixed-methods study [12] was approved by the Harvard Medical School (IRB19-1138) and Chiapas state (5003/8784) institutional review boards and conducted from August 2019 to January 2020. Taking advantage of the pragmatism research paradigm that a mixed-methods methodology provides, we acknowledge health professionals may perceive CPD differently. Therefore, we gathered information from multiple qualitative and quantitative sources to optimize our understanding of their experience [12].

### Qualitative data collection and analysis

Qualitative data was collected via semi-structured interviews, focus group discussions (FGDs), and observation. Participants were recruited using purposeful sampling [13] of all hospital employees, including clinical staff, non-clinical staff, and hospital leadership (ensuring the inclusion of multiple perspectives). Recruitment and data collection were done by MC‑Z, a bilingual healthcare professional and researcher unknown to research participants, who did not undertake any clinical activities locally. Participants were approached, flyers were placed in multiple hospital areas, and the hospital director sent an invitation via email and text message to the entire hospital staff. Verbal consent was obtained for all study participants. An interview guide developed by four researchers (MC‑Z, HG, VD, MIL-M) was piloted with two independent native Spanish-speaking healthcare providers for clarity and coherence (see Electronic Supplementary Material [ESM], part A, “Qualitative instrument”). Semi-structured interviews and focus group discussions were conducted during the same data collection period to ensure research captured both individual and collective information and to accommodate participants’ preferences as they selected which modality they participated in. Interviews and focus groups took place in a private area (classroom) in Spanish by MC‑Z, who audio-recorded and later transcribed and translated them into English in a single-step process.

The primary author (MC-Z) also conducted 40 h of field observations of formal and informal educational activities to understand the context and nuances of information provided by participants. Observation time was distributed across different weekdays and times to capture variation among shifts and days, and field notes with descriptions and reflections were recorded in a journal. These observations were merged with the qualitative and quantitative dataset during the analysis when field notes were iteratively reviewed.

De-identified qualitative data were analyzed using conventional content analysis methods [14]. The dataset was read meticulously. Two authors (MC‑Z, RE-P) open-coded a subset of transcripts to identify key concepts that were developed into a draft codebook that was then revised. The resultant final codebook was subsequently used to code the complete dataset using Dedoose qualitative data management software (SocioCultural Research Consultants, CA, USA). MC‑Z, RE‑P, HG, and VD analyzed the data using an inductive, iterative process in which transcripts and codes were continuously reviewed to develop a set of draft themes; this process continued until saturation was achieved (no new themes were identified). All differences were resolved by the group (MC‑Z, RE‑P, HG and VD) through discussion until consensus was reached. This process yielded seventeen themes that were further refined into a final set of four main descriptive categories.

Throughout the qualitative study phase, reflexivity was maintained by the lead author (MC-Z) through an approach intentionally designed to challenge her own assumptions. While conducting interviews, discussion groups, and translating the data, conscious efforts were made to distinguish her thoughts and hypotheses from the data collected by reviewing field notes and through multiple conversations systematically conducted throughout the data collection stage with VD and MIL‑M, in order to identify and acknowledge assumptions and positionality. The remainder of the research team (a medical anthropologist, health professionals, educators, and researchers) had no relationship with participants or study setting and intentionally left assumptions out while analyzing the dataset. Discussions included acknowledgment and allowance of views, values, and beliefs that may have influenced the analysis.

### Quantitative data collection and analysis

The quantitative portion of the study was designed to allow participants to share their experiences anonymously and to complement qualitative findings. The literature review did not identify any suitable preexisting quantitative instruments for our aims and context. Therefore, a questionnaire was developed following a structured approach described by Artino et al. [15]. Three researchers (MC‑Z, VD, RE-P) with context knowledge and familiarity with the study aims reviewed the qualitative dataset and discussed the most common interview themes that emerged. They subsequently designed potential questionnaire items and multiple-choice questions specific to each one of the constructs to explore. The options for each question were developed based on information shared in the interviews and focus groups (e.g., participants mentioned that the classroom was inadequate for learning, hence that was included as an option when asking about factors limiting participation in CPD activities).

The questionnaire was piloted with four Spanish-speaking health professionals using cognitive interviews to assess the clarity of questions and answers. During the cognitive interviews, we examined thought processes of responses by asking participants to rephrase and explain interpretation of questions. The final version of the questionnaire (see ESM, part B, “Quantitative instrument”) explored four essential constructs within six main questions (48 individual items). The constructs were: (1) perceived importance, (2) motivation (both rated on a 5-point Likert scale), (3) relevant barriers (nine options identified from interviews), and (4) preferred educational strategies (five educational strategies rated on a 1–10 scale). There were 32 educational topics that participants identified as important for future CPD activities included and rated using a 5-point Likert scale (these topics were identified from the qualitative dataset as themes participants mentioned they needed to learn). Content validity was established through an iterative development process plus cognitive interviews. Distribution was done via an anonymous link distributed by mobile message from the hospital director. Demographic data are presented as mean (standard deviation), median (interquartile range), or number (%), and were analyzed using Stata statistical software (Stata Corp, TX, USA).

### Mixed-methods results integration

The quantitative and qualitative datasets were merged to inform the analyses to better understand participants’ experience with CPD activities. Triangulation was conducted using qualitative (transcripts and field notes) and quantitative data to consider different aspects of reality and examine points of convergence or divergence between datasets [13]. Qualitative and quantitative results are reported merged later in this article.

## Results

### Qualitative results

**Fig. 1:**
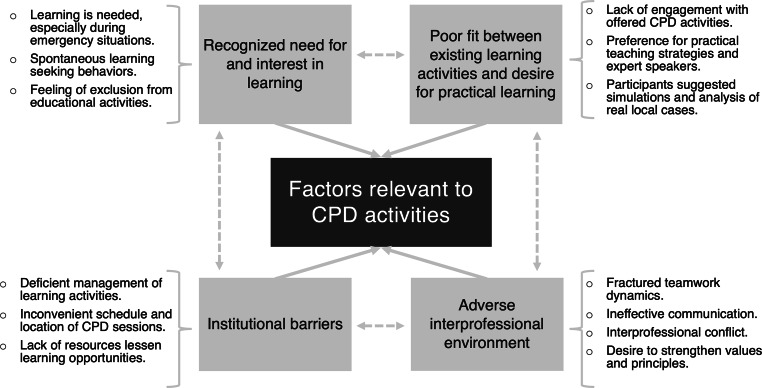
Qualitative results: Factors that impact interprofessional CPD activities in the hospital “Angel Albino Corzo”

Twenty-two semi-structured interviews, three FGDs, and 40 observational hours were conducted. Participant demographic characteristics are described in Tab. 1. From these qualitative data sources, we identified 35 initial codes, and 17 themes that were refined into four main categories (Fig. 1): 1) recognized need for and interest in learning, 2) discrepancy between existing didactic activities and desire for practice, 3) institutional barriers, and 4) adverse interprofessional environment.

**Table 1:** Participant characteristics

	*Interview* *(n* *=* *22)*	*Focus group* *(n* *=* *29)*	*Questionnaire* *(n* *=* *20)*
*Gender, n (%)*			
Female	18 (81.8)	18 (62.1)	16 (80)
Male	6 (27.2)	11 (37.9)	4 (20)
*Profession, n (%)*			
Physician	1 (4.5)	5 (17.2)	6 (30)
Nurse	6 (27.2)	17 (58.6)	14 (70)
Obstetric nurse	2 (9)	3 (10.4)	–
Physician specialist	3 (13.6)	–	–
Allied health professional	4 (18.1)	2 (6.9)	–
Leadership staff	4 (18.1)	2 (6.9)	–
Non-health professional	2 (9)	–	–
*Workplace, n (%)*			
Birth center	5 (22.7)	8 (27.6)	0 (0)
Hospital	17 (77.3)	21 (72.4)	20 (100)

### Quantitative results

Out of 72 eligible hospital employees, 20 completed the questionnaire with only thirteen respondents answering all questions (18% response rate). All respondents were individuals whose primary workplace was the hospital (no responses from the birth center). All respondents considered CPD as important (100%, *n* = 20); 65% (*n* = 13) described it as very important and 35% (*n* = 7) as extremely important. However, only 20% (*n* = 4) felt very frequently motivated to participate in existing CPD activities, 35% (*n* = 7) frequently motivated, 35% (*n* = 7) occasionally motivated, and 10% (*n* = 2) rarely motivated. When questioned about the most important factors that limit participation in CPD, they identified lack of motivation (65%, *n* = 13), inconvenient scheduling of sessions (75%, *n* = 15), an inadequate classroom (65%, *n* = 13), high workload (60%, *n* = 12), unappealing speakers (60%, *n* = 12), and boring sessions (55%, *n* = 11).

Participants rated their preferred learning strategies for future CPD activities on a scale of 1–10. Practical workshops (mean 9.06 ± 1.39), simulations (mean 8.73 ± 1.48), and case analysis (mean 8.44 ± 1.85) received the highest ratings. The questionnaire also explored the topics that health professionals in the HAAC felt were needed in their interprofessional CPD curriculum (results in ESM part C and ESM Fig. S1).

### Mixed-methods integration of results

Overall, the four main qualitative categories were substantiated by participants’ questionnaire responses, as demonstrated in Tab. 2, where we display mixed-methods integration of the results.

**Table 2:** Mixed-methods integration of results: rural health professionals experience with CPD

*Theme*	*Illustrative quote*	*Questionnaire data*	
		*n (%)*	*Response*
Recognized need for and interest in learning	*“We need to learn day by day because day by day things are changing and something new is coming out, diseases that we do not know. And it’s important to learn … because sometimes training can save a person’s life.” *(P2)	20 (100)	Learning is extremely or very important in this hospital
Poor fit between existing learning activities and desire for practical learning	*“They are supposed to be useful, but sometimes the sessions are very tedious. We want to apply the knowledge to practice, we are not only interested in the theory but want to learn how we can use it in everyday life.” *(P1)	13 (65)	Lack of motivation
		12 (60)	Speakers are not appealing
		11 (55)	Sessions are boring
Institutional barriers	*“There are several problems with education in this hospital … First, there is no person in charge of the education department. What can we expect? Moreover, there are failures in the organization … If classes were more regularly performed, we would have more continuing learning. It would help us. (…) I don’t know the reason … everything is abandoned.” *(P10)	13 (65)	The classroom is inadequate
		15 (75)	The schedule is inconvenient
Adverse interprofessional environment	*“Teamwork and communication … that is missing in this hospital. That makes me angry because even though I’ve learned that information, I cannot apply it here in the hospital (…) we are not speaking the same language, even though we are health professionals, we cannot communicate effectively.” *(P10)	10 (85)	Interprofessional teamwork is very/extremely important

#### A. Recognized need for and interest in learning

Participants repeatedly mentioned an interest in learning during interviews and focus groups and all questionnaire respondents considered CPD important (100%, *n* = 20). However, only 20% (*n* = 4) said they felt very frequently motivated to participate in CPD activities. Participants expressed frustration that learning activities were not widely available.* “Training activities … they are scarce. And we are usually excluded from courses in big hospitals. As this hospital is away from the city, we don’t have easy access to training. In the five years I’ve been here I’ve only done 2 courses *(P18)*.”*

Participants report most of their spontaneous learning experiences happened by imitation and as a result of lived experiences, without a recognized formal, institutional approach to continuing education. When participants needed to learn something, they consulted three resources: outdated books and manuals, direct inquiries to colleagues or supervisors, and internet open browser searches on mobile devices. Some described paying for training activities to address their learning needs. *“Here in this hospital, we learn empirically, from experience (…) No one tells us ‘Do this or do that’ … we don’t have formal training and things are done in any way. It doesn’t matter. *(P13)*.”*

Overall, participants described self-doubt regarding their clinical skills and feared that lack of knowledge could harm patients. They asserted that learning could add value to patient care in many situations or save lives, reinforcing their perceived need for continuous learning. *“It was very sad and unfortunate. Losing a patient like that when we know we can prevent it. She was only 24 years old. Yes, if we had more knowledge, we would be able to do many more things to help our patients *(P1)*.”*

#### B. Poor fit between learning activities and desire for practical learning results in disinterest

Individuals described an overall disengagement with educational activities. Traditional lectures (the most frequent method for teaching) were described as *“too theoretical,”* and *“passive.”* Unappealing speakers (60%) and boring sessions (55%) were reported as relevant barriers to engaging with CPD. Sixty-five percent of respondents identified lack of motivation as a relevant barrier to CPD. These cumulative barriers negatively impacted their motivation to attend and organize educational sessions. *“I like it when a session is dynamic and active, not just come and take a sit the entire session. That is boring and annoying … we just sit and listen *(P5)*.” “I was a speaker in a session, and only two doctors attended. I was upset. How could it be that I spend so much time preparing my class and those who need it most, were not there *(P8)*.”*

Participants preferred practical teaching strategies to improve confidence in their daily work, especially if the content reviewed was relevant to real-life activities. They suggested that some teaching strategies such as simulations and real-life case discussions may bring long-term benefits for learning and improved patient care, especially during emergencies. *“I think that it would be an excellent practice if we can have periodic monthly code simulations and get everybody involved. The staff should get used to them … that would help a lot. *(P16)*.”*

#### C. Institutional barriers impact CPD activities

Structural factors limiting effective implementation of CPD in this setting included a lack of human resources, low institutional support for educational activities, limited educational budget, and deficient management processes. *“I think there should be a leader for academic sessions. Because currently, there is no one in charge of these activities, and I don’t think this is working (P22).”*

Participants reported scheduled sessions occurred during their clinical shifts. Time allocated to education was limited due to high administrative burdens and a heavy workload due to staff shortages. In addition, staff had no protected time for CPD activities and prioritized clinical work over education, complicating attendance. If sessions occurred outside of working hours, other barriers like living outside the city prevented attendance. *“There are many colleagues who, even if the session is good, will not attend because it’s far away and especially because of the schedules. Many individuals leave the city or have other jobs and cannot come outside of their working hours *(P4)*.”*

Participants described the lack of a proper setting for educational activities as a key issue for accessing CPD (35%). *“The teaching classroom we are using is not a real teaching classroom. It is small, without enough seats and those available are uncomfortable, the air conditioning does not always work, and if we open the door, we can’t hear the speaker *(P22)*.”*

Additional institutional barriers include equipment shortages that may impact learning; for example, a nurse interested in learning about manual ventilation devices was unable to do so because no devices were available.

#### D. Adverse interprofessional environment

While interviews focused primarily on education, participants also described contextual factors that shaped their professional and learning environment, including an absence of collaboration and teamwork, and ineffective communication in their workplace. *“This team is so fractured … doctors believe they are an exclusive group! They believe that nurses don’t have the intellectual capacity to attend a certain course with them, that they don’t have the needed academic skills, and that they don’t understand *(P10)*.”*

A lack of communication between health professionals that disrupted workflow and involvement in interprofessional educational activities was widely described. Several interviewees explained that miscommunication limited their participation in sessions because they were not notified of learning sessions or were uninformed of unexpected schedule changes.

*“Sometimes we don’t communicate at all even when we know the information … For example, this morning, we had a surgery scheduled, and it was cancelled because we have no blood … we knew that since yesterday. But the patient was admitted because the night staff did not know! *(P1)*”.*

Conflict among health professionals working together was frequently described in clinical practice and during interprofessional education (IPE) sessions resulting from a misunderstanding of each other’s roles. This lack of understanding between distinct health professionals compromised patient care performance and limited the effectiveness of interprofessional activities. *“The staff is having problems between them, they don’t support each other, and the work is never done at 100%. There is always the discussion about this is not my job, this is your job, and time is wasted. They look after their own interests only, not after the patients *(P9)*.”*

## Discussion

This study describes health professionals’ perceptions of CPD activities in a rural healthcare context and identifies the challenges impacting healthcare providers’ learning. All the participants recognized the importance of CPD and demonstrated a desire for learning. However, the existing CPD activities did not satisfy their perceived needs and, therefore, a widespread lack of engagement hindered CPD initiatives. This is a critical contradiction that may come from the multiple challenges to participating in continuous learning activities and from the type of learning opportunities currently available.

Engagement with CPD arises from a combination of attitudinal and behavioral factors including attention, intrinsic motivation, cognitive and physical energy, and perceiving learning as purposeful [16]. A misalignment in these factors most likely contributed to the low engagement found in this study, where perceived control and choice over learning activities were very low. Multiple frameworks aligned with andragogical principles could support efforts to increase lifelong learners’ engagement in CPD in this setting (e.g., self-directed learning, self-determination theory, situated learning, and workplace affordances) [17].

We found that the current methods used in educational activities hampered learners’ engagement and learning as the traditional lecture style sessions did not satisfy the desire for practical learning and hands-on approaches to CPD. Passive learning was the predominant method of instruction in this setting, and we found strong interest among learners and leadership to transition to active approaches to education. Active learning has been proven to increase the effectiveness of learning in a broad range of professions [18] and it has been implemented in rural areas with positive results in learners’ engagement [19]. Participants described clinical simulation or case-based learning as potential solutions to poor engagement. Although implementing simulation programs in low-resource settings is difficult, it has shown encouraging results [20, 21] and a shift from lecture-based educational activities to an active learning model could potentially support learner engagement in this setting.

Other perceived challenges to CPD were related to the institutional approach to managing educational activities (deficiencies in leadership, faculty, and material resources) that may be analogous to those observed in similar rural settings. In Australia, consistent with our findings, Gum and colleagues found that time constraints, working in silos, limited communication, poor sense of belonging, misconceptions about professional roles, and interprofessional conflict contributed to reluctance in engaging in learning activities [9].

Rural healthcare workforce individuals need interprofessional and reliable formal and informal CPD opportunities to address the diverse challenges in this context. Utilizing a learning organization approach in which health professionals have plenty of explicit and implicit opportunities to address their learning needs could be an effective method of increasing CPD engagement [16]. Resources and dedicated personnel allocated to CPD are critical to provide opportunities to overcome the existing multifactorial barriers. Our findings are relevant to health professions educators implementing CPD curricula in similar locations and highlight the importance of anticipating challenges and developing strategies to overcome them.

Importantly, the disruption generated by the COVID-19 pandemic may have had a great impact on CPD in this and other rural settings where healthcare professionals were already strained to provide optimal care with limited resources [22]. However, the pandemic also highlighted the key role of continuing education in health care. Skalr et al. described a framework that may support future CPD efforts and could help address some of the challenges found in the present study (i.e., partner with academic health centers, improve workplace-based learning, enhance assessment and feedback for learning, and promote a culture of continuous learning) [23].

The influence of a rural context on the learning environment and educational strategies is not fully understood, and further research is needed to develop approaches to decreasing the barriers to CPD activities in these settings. A multicentric study could further help characterize variations and similarities across different regions and support the development of strategies to effectively address these barriers that are potentially generalizable and scalable.

### Limitations

This study was conducted at a single healthcare facility, and our results may not be generalizable to other facilities or regions. However, the major findings of this study may be relevant for similar settings and could be used as an initial framework for future research.

The qualitative data collection was conducted during a pre-defined 5‑week period entirely by one author, potentially introducing bias. This is an intrinsic situation in qualitative research and intentional efforts were carried out to maintain reflexivity. The quantitative portion of our study faced several limitations, including small sample size and a response rate lower than expected. Likewise, evaluation of the psychometric properties of this instrument may be required for future use. Birth center staff were not included in the questionnaire due to internal communication issues (illustrating some of the administrative and communication challenges that CPD faces in this setting). We do not have demographic information for non-responders, and it was unclear why seven individuals left questions unanswered. Therefore, our quantitative results might not accurately represent the study population perceptions. However, responders were proportionally representative of the demographic distribution of the hospital staff, and we found convergence between the identified themes and the questionnaire data, suggesting that survey respondents had similar opinions to those collected in the qualitative data.

## Conclusions

Health professionals working in rural settings face multifactorial challenges preventing them from effectively conducting CPD activities despite their great interest. A strong inclination for context-specific active learning strategies hampered by current educational methods and institutional factors highlighted the need to implement CPD activities that satisfy individuals’ needs. Furthermore, strategies to strengthen interprofessional continuing professional development may enhance not only learning but also improve collaborative care in this setting but must be carefully planned and supported by local, regional, and national leadership. These findings could inform efforts to strengthen CPD in other rural contexts, but the many factors unique to this context must be taken into consideration. Rigorous multicentric research exploring evidence-based educational strategies to address the specific lifelong learning challenges health professionals face in the rural context is needed.

## Supplementary Information

The Electronic Supplementary Material (ESM) contains the qualitative (semi-structured interview guide) and quantitative (questionnaire) instruments used in this study. Moreover, we provide one table with supplementary results (with the learning needs identified by participants) and a figure with the highest rated topics for future CPD curriculum as they could be useful for educators planning a rural CPD curriculum.
