# Supplementary material for: Continuing professional development challenges in a rural setting: A mixed-methods study
Source: Perspect Med Educ. 2022 Aug 9;11(5):273–80. doi: 10.1007/s40037-022-00718-8 (PMC9360663; doi:10.1007/s40037-022-00718-8)

# Electronic Supplementary Material (ESM)

# Qualitative instrument: Sample of semi-structured interview guide (Clinical staff)

1. I’m interested in understanding what learning looks like in this hospital. Can you describe for me the most recent situation when you felt you learned something?

[Probes]Who___? What___? How___?

1. I know that some lectures and workshops have been conducted in this hospital in the past. Can you describe for me what was positive about participating in them? What could have been improved or changed?
2. Now can you think about a time when you needed to research information in order to help a patient. Can you tell us how you searched for that information?

[Follow up] Can you describe for us how it happened or provide an example?

[Follow up] What other resources have you ever used for this purpose?

1. Can you tell me what you think about your own continuous learning process as a health professional? Is it important, not important, why? [Probe] Example __________
2. After all we’ve discussed today, I have some questions about what you think would help you learn better.

What suggestions would you make about scheduling an educational activity?

What suggestions would you make about format?

How about resources?

1. I’m interested in your recommendations about topics that would be useful to you professionally. Can you please list all the things you’d like to learn?

[Probe until list is exhausted]

1. Is there any other suggestion regarding the learning activities we will develop for this hospital that you would like to share?

# Quantitative instrument: Questionnaire.

Thank you for filling out this form. This anonymous questionnaire will help us to understand your experience with interprofessional continuous professional development activities in the hospital and to know your preferences for future educational activities. It will take less than 10 minutes to complete this form.

1. How important is the continuing professional development process for you in this hospital?
   1. Not important
   2. Slightly important
   3. Moderately important
   4. Very important
   5. Extremely important
2. How frequently you feel motivated to participate in educational activities in this hospital?
   1. Very frequently
   2. Frequently
   3. Occasionally
   4. Rarely
   5. Never
3. What are the factors that limit your participation in continuous professional development activities in this hospital? Please select YES in the options that are most important to you.

| *Yes* | *No* |
| --- | --- |

- 1. The workload is too high
  2. The schedule of classes is not convenient for me
  3. The sessions I’ve seen are boring
  4. I don’t know when sessions are
  5. Too few people attend to the sessions
  6. I don’t feel motivated to attend
  7. The classroom is inadequate
  8. I don’t feel comfortable speaking during the sessions
  9. The speakers are not appealing

1. **How would you like to learn?** Please tell us how much you would like each of the following teaching methods to be included in the academic plan of this hospital. Select 1-10 according to the scale provided:

| *Definitively should NOT*  *be included* | | | | | | *Definitively should*  *be included* | | | | | |
| --- | --- | --- | --- | --- | --- | --- | --- | --- | --- | --- | --- |
| *1* | *2* | *3* | *4* | *5* | *6* | | *7* | *8* | *9* | *10* |  |

- 1. Lecture (Theory based)
  2. Case analysis
  3. Video based session
  4. Simulations
  5. Practical workshop

1. We are trying to evaluate which topics are important for you to include in future educational activities in this hospital. The following set of questions will explore how important some topics are for you. **How important do you consider each of the following topics for YOUR learning?**

|  | Not at all important | Slightly important | Moderately important | Very important | Extremely important |
| --- | --- | --- | --- | --- | --- |
|  |  |  |  |  |  |
| 1. Obstetric emergencies: Hemorrhage and Preeclampsia |  |  |  |  |  |
| 1. Abortion management |  |  |  |  |  |
| 1. Obstetric care (Birth surveillance and complications) |  |  |  |  |  |
| 1. Preterm birth |  |  |  |  |  |
| 1. Family planning methods |  |  |  |  |  |
| 1. Respectful birth |  |  |  |  |  |
|  |  |  |  |  |  |
| 1. Crisis resource management in critical patients |  |  |  |  |  |
| 1. Cardiopulmonary Resuscitation |  |  |  |  |  |
| 1. Trauma patient care |  |  |  |  |  |
| 1. Sepsis patient management |  |  |  |  |  |
| 1. Airway management |  |  |  |  |  |
| 1. Acute cardiomyopathy |  |  |  |  |  |
| 1. Stroke |  |  |  |  |  |
| 1. Poisoning management |  |  |  |  |  |
| 1. Bites and stings |  |  |  |  |  |
|  |  |  |  |  |  |
| 1. Newborn care (term and preterm) |  |  |  |  |  |
| 1. Pediatric care (Emergency and routine care) |  |  |  |  |  |
| 1. Diarrhea, dehydration, and liquids management |  |  |  |  |  |
|  |  |  |  |  |  |
| 1. IV access |  |  |  |  |  |
| 1. Sutures |  |  |  |  |  |
| 1. Bandages and immobilization |  |  |  |  |  |
| 1. Drug dilution and administration |  |  |  |  |  |
| 1. Cardiotocography |  |  |  |  |  |
| 1. OR processes and techniques |  |  |  |  |  |
|  |  |  |  |  |  |
| 1. Information search and critical analysis strategies |  |  |  |  |  |
| 1. Effective communication techniques |  |  |  |  |  |
| 1. Interprofessional teamwork |  |  |  |  |  |
| 1. Leadership and professionalism |  |  |  |  |  |
| 1. Professional Identity |  |  |  |  |  |
| 1. Palliative care |  |  |  |  |  |
| 1. Patient safety |  |  |  |  |  |
| 1. Work stress |  |  |  |  |  |

1. Is there any other topic that you consider EXTREMELY important for YOUR learning in this hospital? ______________________________________________________________________

# Supplementary results

| **Supplementary Table 1.** Learning needs identified by interview participants. | | | | | | | | | |
| --- | --- | --- | --- | --- | --- | --- | --- | --- | --- |
|  | Specific topic | Mentions | | Clinical staff (n) | | | | Non-clinical staff (n) | |
|  |  | **n** (24) | % | Nurse | ObN | GP | Sp. | Other | Leader |
| 1 | Obstetric emergencies (Hemorrhage and Preeclampsia) | **18** | 75% | 6 | 2 | 2 | 2 | 3 | 3 |
| 2 | Interprofessional teamwork | **18** | 75% | 6 | 2 | 2 | 2 | 2 | 4 |
| 3 | Obstetric care (Birth surveillance and complications) | **17** | 71% | 5 | 2 | 1 | 3 | 2 | 4 |
| 4 | Crisis management in critical patients | **17** | 71% | 6 | 1 | 1 | 3 | 3 | 3 |
| 5 | Effective communication techniques | **13** | 54% | 5 | 2 |  | 1 | 2 | 3 |
| 7 | Newborn care (term and preterm) | **12** | 50% | 3 | 1 | 1 | 2 | 2 | 3 |
| 8 | Conflict management | **11** | 46% | 3 |  | 1 | 1 | 3 | 3 |
| 9 | Leadership and professionalism | **9** | 38% | 3 | 1 | 1 | 1 | 1 | 2 |
| 10 | Trauma patient care | **8** | 33% | 3 |  | 1 | 1 | 2 | 1 |
| 11 | Pediatric care (Emergency and routine care) | **8** | 33% | 4 |  | 1 |  | 1 | 2 |
| 12 | Cardiopulmonary Resuscitation | **7** | 29% | 2 |  | 1 | 2 | 2 |  |
| 13 | Information search techniques | **6** | 25% | 3 | 1 | 1 | 1 |  |  |
| 14 | Clinical record management | **5** | 21% | 2 |  |  | 1 | 1 | 1 |
| 15 | Hypertension and diabetes care and acute complications | **5** | 21% | 3 | 1 |  |  |  | 1 |
| 16 | Work stress | **5** | 21% | 3 | 1 |  |  |  | 1 |
| 17 | Patient safety | **5** | 21% | 1 |  |  |  | 1 | 3 |
| 18 | Drug administration, dilutions and IV access | **4** | 17% | 2 |  |  |  | 1 | 1 |
| 19 | Airway management | **4** | 17% | 1 |  | 1 | 2 |  |  |
| 20 | Respectful birth | **4** | 17% | 2 | 1 |  |  |  | 1 |
| *ObN: Obstetric nurses. GP: General practitioner. Sp. Physician specialist. | | | | | | | | | |

**ESM Fig. S1** Highest rated topics by health professionals for future CPD curriculum


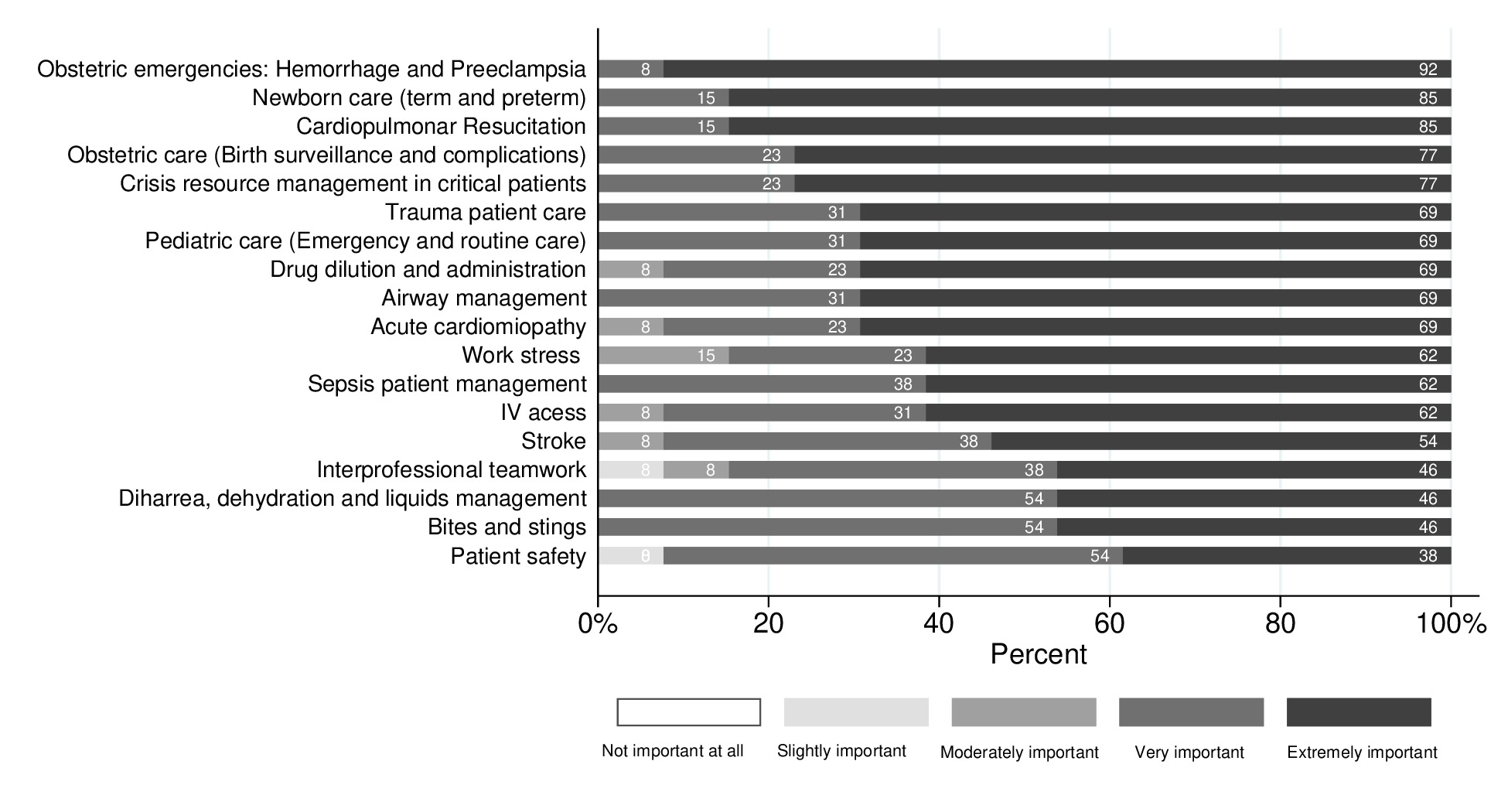

Supplement: Supplementary file 1 — The Electronic Supplementary Material (ESM) contains the qualitative (semi-structured interview guide) and quantitative (questionnaire) instruments used in this study. Moreover, we provide one table with supplementary results (with the learning needs identified by participants) and a figure with the highest rated topics for future CPD curriculum as they could be useful for educators planning a rural CPD curriculum. [file 40037_2022_718_MOESM1_ESM.docx]
